# Recent Advances in ^19^Fluorine Magnetic Resonance Imaging with Perfluorocarbon Emulsions

**DOI:** 10.15302/J-ENG-2015103

**Published:** 2016-03-16

**Authors:** Anne H. Schmieder, Shelton D. Caruthers, Jochen Keupp, Samuel A. Wickline, Gregory M. Lanza

**Affiliations:** 1 Division of Cardiology, Washington University School of Medical, St. Louis, MO 63110, USA; 2 Toshiba Medical Research Institute USA, Inc., Cleveland, OH 44143, USA; 3 Department of Biomedical Engineering, Washington University, St. Louis, MO 63130, USA; 4 Philips Research Hamburg, Hamburg 22335, Germany

**Keywords:** fluorine, magnetic resonance imaging (MRI), dual-tuned coil, perfluorocarbon, angiogenesis, cell labeling

## Abstract

The research roots of ^19^fluorine (^19^F) magnetic resonance imaging (MRI) date back over 35 years. Over that time span, ^1^H imaging flourished and was adopted worldwide with an endless array of applications and imaging approaches, making magnetic resonance an indispensable pillar of biomedical diagnostic imaging. For many years during this timeframe, ^19^F imaging research continued at a slow pace as the various attributes of the technique were explored. However, over the last decade and particularly the last several years, the pace and clinical relevance of ^19^F imaging has exploded. In part, this is due to advances in MRI instrumentation, ^19^F/^1^H coil designs, and ultrafast pulse sequence development for both preclinical and clinical scanners. These achievements, coupled with interest in the molecular imaging of anatomy and physiology, and combined with a cadre of innovative agents, have brought the concept of ^19^F into early clinical evaluation. In this review, we attempt to provide a slice of this rich history of research and development, with a particular focus on liquid perfluorocarbon compound-based agents.

## 1 Introduction

Although hydrogen-based (^1^H) magnetic resonance imaging (MRI) predominates over the cadre of magnetic resonance (MR) techniques employed clinically, renewed interest in ^19^fluorine (^19^F) MRI continues to increase, particularly for molecular imaging applications using perfluorocarbons (PFCs) as a fluorine source. MR fluorine spectroscopy and imaging dates back to 1977, a time when human ^1^H MRI was in its infancy [1]. Several investigators contributed to the early technical foundation of ^19^F imaging [2–7].

### 1.1 Why ^19^fluorine?

Interest in ^19^F nuclei imaging reflects its potential as a quantitative MRI contrast agent. ^19^F has 100% natural abundance, a spin of 1/2, and a gyromagnetic ratio of 40.08 MHz·T^−1^ (slightly lower than the 42.58 MHz·T^−1^ of ^1^H), resulting in 83% of the sensitivity of ^1^H [8, 9]. With seven outer-shell electrons, ^19^F chemical shifts (CSs) are more sensitive to the local environment than ^1^H with its single electron. Indeed, the spectroscopic signatures of ^19^F compounds can vary over a range more than 200 ppm [10, 11], offering the potential for definitive identification of many compounds even at lower clinical field strengths. Although soft body tissues, which contain 55%–75% water, contribute a substantial mobile ^1^H signal, fluorine is essentially absent from soft tissues and is only found immobilized in bones or teeth, where its very short spin-spin relaxation time (T2) renders ^19^F virtually invisible to conventional MR techniques. ^19^F MRI of high-density exogenous fluorine compounds accumulating at target sites offers high contrast-to-noise ratios (CNRs) and improved quantification potential. Furthermore, the negligible ^19^F background obviates the use of the serial pre-/post-contrast image comparisons that are requisite for differentiating superparamagnetic (e.g., iron oxide) or paramagnetic metal (e.g., gadolinium) contrast from the background in most molecular imaging studies.

### 1.2 Perfluorocarbons (PFCs)

PFC nanoparticles are 98% PFC by volume, which for perfluorooctylbromide (PFOB, 1.98 g· mL^−1^, 498 Daltons) equates to a fluorine concentration of approximately 100 mol· L^−1^ [12]. PFC nanoparticles are distinctly different from other oil-based emulsions by virtue of the physical-chemical properties of fluorine, the most electronegative of all elements, and the unique properties of C—F bonds [13]. The fluorine-substituted hydrogen in perfluorochemicals creates bulkier, stiffer compounds that typically adopt a helical conformation with the molecules chemically distinct but often closely intertwined [13]. The C—F bond is chemically and thermally stable and its dense electron cloud creates a barrier to encroachment by other chemical reagents, rendering it virtually non-chemically reactive [13]. The resultant large surface area combined with the low polarizability of the fluorinated chains enhances hydrophobicity. Interestingly, PFCs are both hydrophobic and lipophobic!

The biocompatibility of liquid fluorocarbons is well documented in numerous preclinical animal studies. Even at large doses, most fluorocarbons are innocuous and physiologically inactive. No toxicity, carcinogenicity, mutagenicity, or teratogenicity effects have been reported for pure fluorocarbons within the 460–520 MW range. PFCs have tissue half-life residencies ranging from 4 days for PFOB up to 65 days for perfluorotripropylamine, and are not metabolized; rather, they are slowly reintroduced to the circulation in dissolved form by lipid carriers and expelled through the lungs. However, increased pulmonary residual volumes from expired PFC, which is denser than air, and reduced pulmonary compliance, is noted with blood-transfusion-level dosages, particularly with some early-generation PFC emulsions in rabbits, swine, and macaques, although not in mice, dogs, or humans [13–15]. Similarly, PFC nanoparticles are cleared through the monocyte-phagocyte system (MPS), previously referred to as the reticulo-endothelial system (RES). Repeated high dosages of PFCs by these cells can lead to the release of macrophage cytokines, resulting in flu-like symptoms [12]. Moreover, acute engorgement of the liver with PFC nanoparticles due to a large-volume infusion or repeated high-volume dosing can result in the transient physical compression of tissue with mild hepatocellular damage, resulting in reversible elevations in serum transaminases [16].

Furthermore, it must be recognized that in rodents, nanoparticles such as PFC nanoparticles can pass rapidly and directly into the biliary system, where they flow into the small intestine [17, 18]. In non-rodent species, such as rabbits or humans, this rapid bioelimination is not present [19]. Thus, pharmacokinetics, biodistribution, and the safety of PFC nanoparticles will differ significantly between rodent and non-rodent species, affecting the dosages administered to compensate for the loss or the assessments of dose-safety margins of particles and their contrast or therapeutic payloads [18].

Early preclinical and later clinical research with PFCs involved liquid breathing, recognizing the high oxygen-dissolving capacity of PFCs and the need to address surfactant deficiencies in preterm babies [20–25]. Although it was an interesting and effective application, this use of PFCs was rapidly superseded by the development of alternative surfactant replacement technologies. However, the oxygen-dissolving capacity of PFC emulsions was not forgotten, and efforts to develop these agents as artificial blood substitutes were pursued with limited success.

Fluosol-DA (Green Cross, Japan) was the first PFC emulsion approved for blood replacement, but it was associated with significant hemodynamic compromise related mostly to the choice of surfactant [26–32]. A similar particle, Fluosol-43, was later developed, substituting an albumin surfactant to counter the unstable hemodynamic issues. Fluosol-DA was composed of a mixture of perfluorodecalin (PFD) and perfluorotributylamine, which are fluorine compounds with complicated MR spectra. From an MR cell-tracking perspective, PFD offers limited imaging or spectroscopy potential, even with today’s more highly refined instruments and techniques [33, 34]. The next generation of PFCs pursued clinically for artificial blood substitutes were PFOB (Alliance Pharmaceuticals) and perfluorodichloro-octane (PFDCO, HemaGen/PFC, Inc.). These fluoro-compounds reduced pulmonary gas stacking and decreased the time for PFC bioelimination from tissues. These second-generation PFC-based blood substitutes utilized phospholipid surfactants for better biocompatibility, better oxygen-dissolving capacity, and fewer significant side effects, improved bioelimination rates, and were amenable to large-scale commercial production. The oxygen dissolved in the PFC liquid was easily extracted by oxygen-deprived tissues. However, the oxygen-loading capacity of PFCs is linearly related to the partial pressure of oxygen in equilibrium with the emulsion. PFCs demonstrate a nearly flat, linear oxygen dissociation curve in contrast to the sigmoidal dissociation curve of hemoglobin. As a result, most of the oxygen dissolved in the PFC is released in the high-pressure atmosphere of the arteries, with little oxygen being available for the capillary network where the partial pressure of oxygen is lower and the need is greatest. PFC products essentially failed clinically as blood substitutes.

During this time, Dr. Robert Mattrey, working with the Alliance PFOB emulsion platform, conducted early studies to determine whether these particles offered clinical imaging potential with ultrasound (US), computed tomography (CT), and MRI [35–44]. Although much of this work centered upon liquid PFC nanoparticles for US and CT and required large volumes of materials to provide blood pool contrast, only the use of PFOB emulsions for gastrointestinal (GI) contrast (negative contrast) with ^1^H MRI gained traction. In this application, PFOB particles offered significant imaging and procedural advantages over standard barium contrast studies, but at a higher cost. The much lower cost of using barium for GI imaging won out.

## 2 MRI with PFC emulsions

The stability and significant prior human experience with PFOB offered a unique modifiable nanoparticulate theranostic platform technology that has been extensively exploited in a variety of medical applications by our laboratory, both alone and in collaboration with many others [16, 45–79]. For ^1^H MRI, PFC nanoparticles provided a stable platform for high payloads of lipophilic gadolinium chelates to enhance targeted MR molecular imaging. However, with increasing concerns regarding gadolinium-induced nephrogenic systemic fibrosis and acute complement activation noted in clinical trials [80–82], ^19^F imaging with targeted PFOB nanoparticles at 3 T was reconsidered in order to address these significant unmet clinical needs.

More recently, MR cell tracking with fluorocarbon labeling was pursued as an alternative to more commonly used iron oxide nanoparticles [83–90]. These investigators brought increased attention to the use of cyclic perfluoro-15-crown-5-ether (PFCE) and linear perfluoropolyether (PFPE) molecules that have repeating —CF_2_CF_2_O— units for improved ^19^F signal-to-noise ratio (SNR) and detectability [63, 91–101]. When used for systemically targeted molecular imaging, the heavy accumulation of PFCE in the liver and spleen combined with its very prolonged biological clearance rate presented a noteworthy human safety barrier to clinical translation. In the context of cell tracking, the PFC emulsion particles are internalized by cells *ex vivo* then administered, often locally by image-guided injection, and the vast excess of PFC particles associated with systemically injected PFC emulsions is avoided. In this context, the prolonged duration of these compounds might be considered an asset for the serial cell tracking over a more prolonged timeframe. More recently, PFPE has gathered increased interest with the advent of cell tracking, with its > 40 chemically equivalent fluorine atoms simplifying the ^19^F nuclear magnetic resonance (NMR) spectra, and a small T1/T2 ratio. Unusually for a PFC, the end-groups may be chemically functionalized, such as with dyes [92, 102].

## 3 ^19^F detectability with MR

Since the concentrations of PFCs employed in biological systems are low, the typical ^19^F imaging SNR is low compared with that of ^1^H MRI. While some applications do administer large volumes of highly fluorinated fluoro-compounds and produce effective ^19^F images, high doses (more than 1–5 g/kg for PFCs) may be limited in humans by the aforementioned safety concerns [12]. To date, magnetic resonance spectroscopy (MRS) studies in a whole-body scanner suggest a minimum detectable limit of ^19^F at 1.5 T of about 30 μmol.g^−1^ wet weight in a volume of about 33 mL in 6 min, based on surface coil measurements following the infusion of 5-fluorouracil (5-FU) chemotherapy [103]. At higher field strengths, spectroscopic 5-FU quantification of about 5 nmol.g^−1^ in 0.5–1.2 g samples at 11.7 T and one-hour data acquisition is possible [104].

PFCE contains 20 resonant equivalent ^19^F nuclei that enrich the signal to allow 30 μmol· g^−1^ wet weight detection in a 4-min scan time in a volume of 60 μL *in vivo* at 9.4 T [67]. Related measurements by Partlow et al. [63] suggested a detection limit (SNR > 3) of approximately 2000 cells for PFCE, approximately 10 000 cells for PFOB-labeled cells for *in vitro* with ^19^F MRS (11.7 T), and approximately 6000 PFCE-labeled cells/voxel *in vitro* with ^19^F MRI (11.7 T). For imaging at 1.5 T, the *in situ* localized injection of 4 million CE-labeled stem cells produced a strong fluorine signal. Quantification of PFC intracellular content following 12-hour cellular co-incubation without adjunctive transfection agents reached up to 3 pmol per cell. Moreover, the r1 contrast sensitivity of PFCE, like that of other PFCs, improves with increasing magnetic field strength, which has proved advantageous, as clinical MR instrumentation has increased from 3 T to 7 T and above. However, it also means that non- or off-target ^19^F noise has become proportionately more prominent. Clearly, PFC particles markedly enhance the local fluorine concentration to permit cell tracking or targeted molecular imaging studies.

Further ^19^F signal detectability at clinical field strengths (3 T and below) can be achieved by incorporating a relaxation-modifying agent, lipid-anchored-gadolinium (Gd^3+^), into the outer surfactant layer surrounding PFC nanoparticles. Neubauer et al. [105] showed that the addition of lanthanide imparted a four-fold augmentation of the ^19^F signal from 200 nm PFC nanoparticles at 1.5 T. This was reflected as a 125% increase in contrast signal when the nanoparticles were targeted to fibrin clots *in vitro*. These investigators also noted that modifications of the Gd^3+^ surface concentration and the lanthanide metal position relative to the particle surface could be used to tailor or eliminate the ^19^F benefit. This concept was corroborated by Harvey et al. [106], and then further explored by de Vries et al. [107]. de Vries et al. studied this concept using three different PFC emulsions at five magnetic field strengths. Using PFCE and three lipophilic chelates (Gd^3+^ DOTA–DSPE, Gd^3+^ DOTA–C6–DSPE, and Gd^3+^ DTPA–BSA), they noted that Gd^3+^ DTPA–BSA, which positions the lanthanide metal close to the PFC core, had a strong influence on ^19^F R1 compared with Gd^3+^ DOTA–DSPE and Gd^3+^ DOTA–C6–DSPE, which position the metal further into the surrounding water and away from the PFC core. At typical clinical field strengths (1.5–3.0 T), Gd^3+^ DTPA–BSA inclusion favorably influenced ^19^F R1, but at higher field strengths (6.3–14 T), supplemental Gd^3+^ did not lead to an improvement and could adversely increase ^19^F R2. Because ^19^F r1 relaxivity inherently improves with increasing field strengths, as used in preclinical animal scanners, the need to further augment the ^19^F signal with a lanthanide has diminished.

## 4 MR ^19^F coils

MRI of fluorinated contrast agents is challenged by low sensitivity, especially at clinical field strengths (3 T or below). ^19^F molecular imaging has been enabled by the effective concentration of PFC probes to specific pathological sites via ligand-directed homing; the image-guided local injection of cells enriched with high PFC payloads into regions of interest; the use of single resonance ^19^F molecules; and the correction of CS artifacts [108], ultrafast MR [109–111], and compressed sensing [100] acquisition techniques for multispectral agents. However, clear improvements in radiofrequency (RF) coil design, and particularly the development of dual ^19^F/^1^H imaging, have emerged as essential advancements to ^19^F imaging technology [73, 112–118].

Single-frequency-mode RF coils for ^19^F/^1^H MRI are typically employed where the coil frequency is tuned to either ^19^F or ^1^H frequency to maximize the SNR. This approach requires time and potentially leads to coil motion and displacement that can complicate later ^19^F/^1^H image co-registration. Two-coil approaches such as a volume coil for ^1^H imaging and a surface coil for ^19^F imaging can be effective for acquiring the individual signals, but their inherently different sensitivity patterns can lead to image discordance when the two frequency datasets are combined. Auto-tuned RF coils that use an external computer program emerged to switch resonant frequency are an option in some of the more expensive imaging systems, but these come with increased complexity [119]. Overall, single-frequency-mode RF coils for ^19^F/^1^H MRI imaging are challenged to overcome several issues: diminished SNR, differential *B*_1_ field inhomogeneity artifacts, and co-registration error of ^1^H/^19^F signals.

To address these challenges, dual ^19^F/^1^H coils were explored. Dual-frequency coils for two well-separated resonant frequencies such as ^1^H (42.58 MHz·T^−1^) and ^13^C (10.71 MHz·T^−1^) are typically designed utilizing shunting and multiple pole methods. Unfortunately, this classic approach is less suited for ^19^F/^1^H MRI coils where the ^19^F gyromagnetic ratio of 40.08 MHz·T^−1^ is close to that of ^1^H. One design approach considered a universal matching circuit via multiport input for multi-frequency. Another tactic considered close dual-frequency coil strategies based on the special resonant property of a birdcage resonator, particularly for preclinical scanners, wherein two crossed cages or two different modes of a birdcage are used to achieve the double resonance.

**Figure 1:**
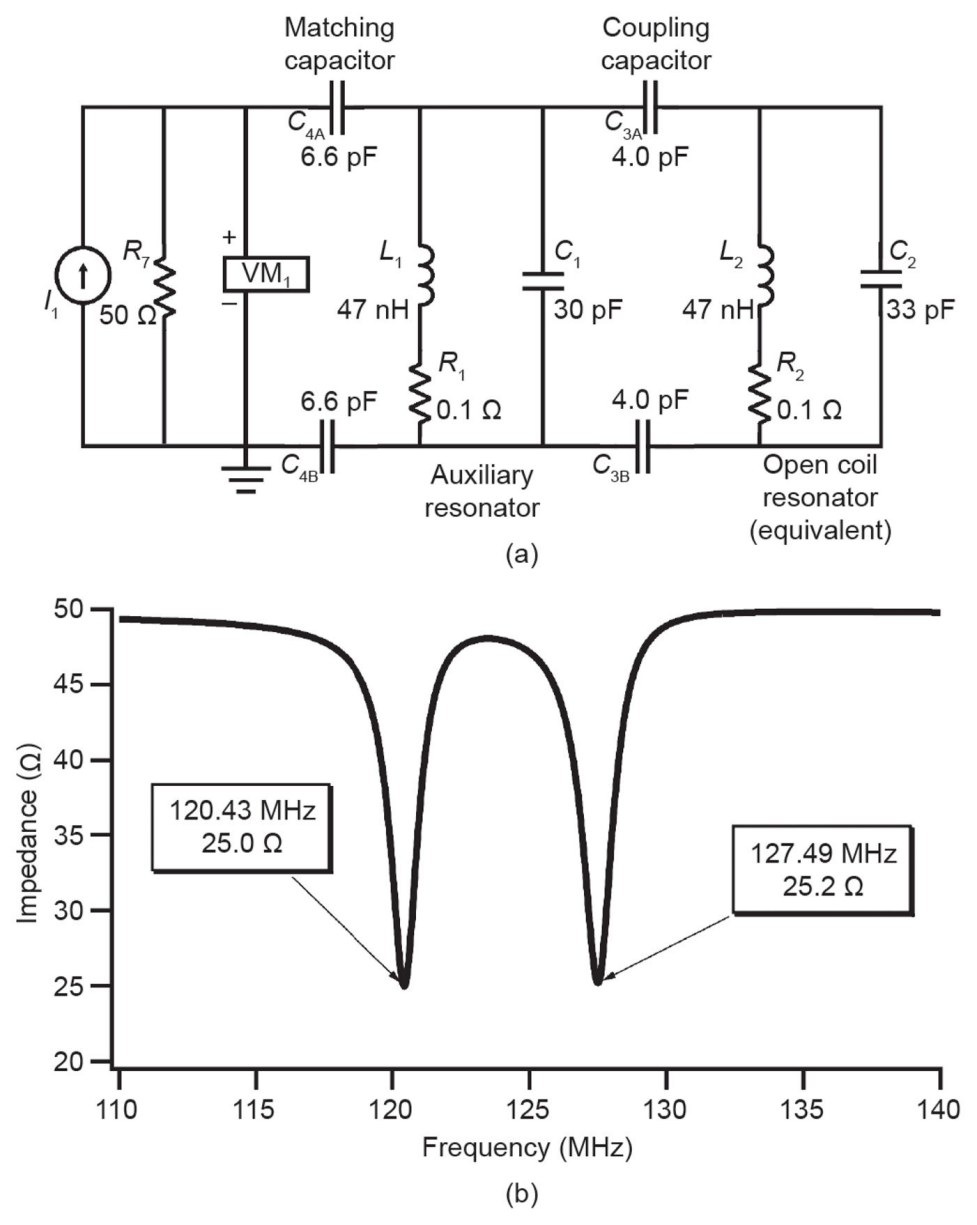
The dual-frequency coil is conceptualized as two separate L CR resonators (*L*_1_, *C*_1_, *R*_1_) and (*L*_2_, *C*_2_, *R*_2_) electrically coupled via capacitor *C*_3_ (a) The equivalent circuit diagram; (b) the impedance magnitude output of a SPICE simulation of two capacitively coupled resonators. ^19^F is 120.43 MHz and ^1^H is 127.49 MHz at 3 T. Reproduced with permission from Ref. [118].

**Figure 2:**
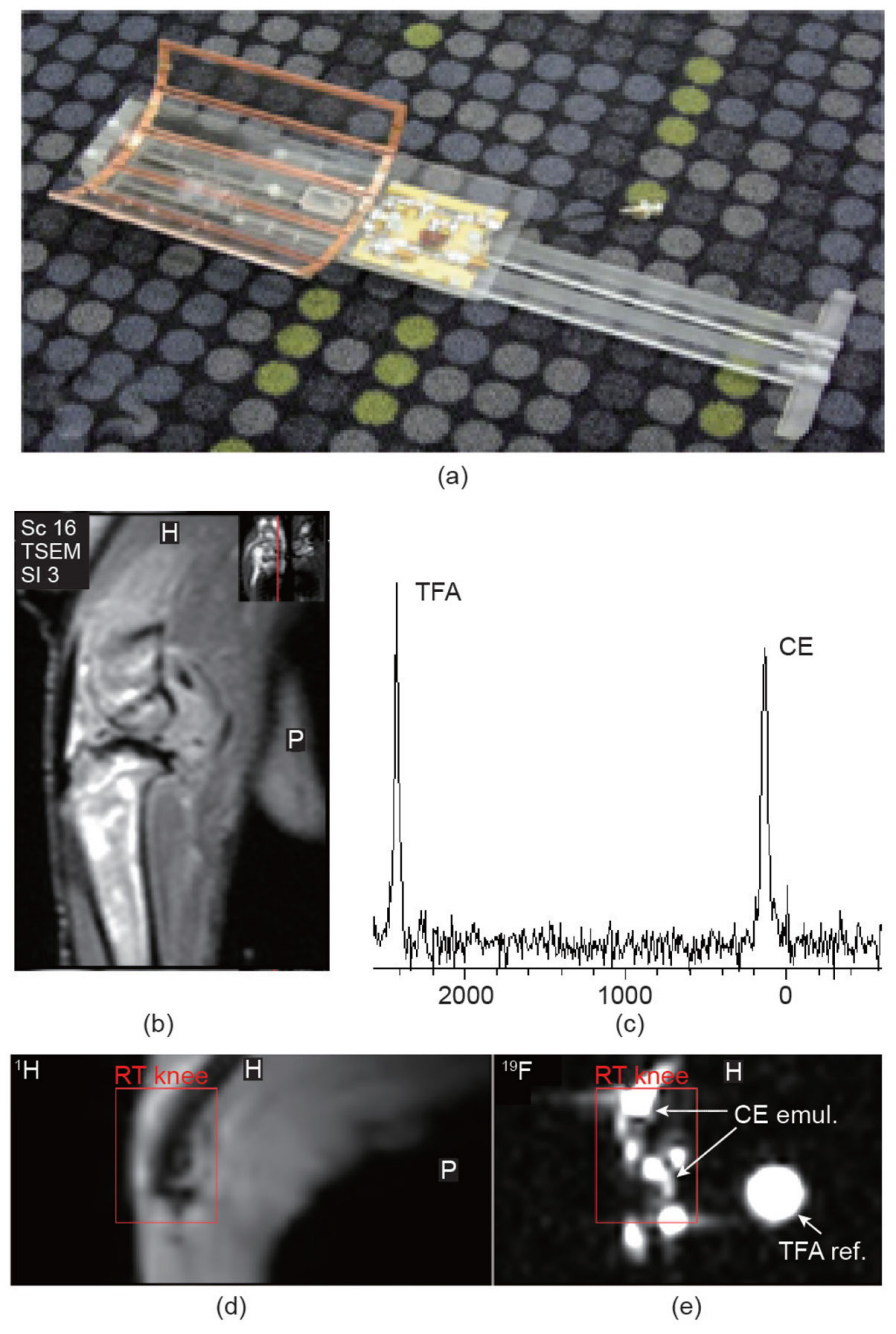
(a) Photograph of completed dual-frequency coil and supporting electronics. The six-leg (five-loop) coil structure is shown in the upper left portion of the image. (b) High-resolution ^1^H image of an arthritic rabbit knee, highlighting anatomic features. (c) Spectral lines corresponding to the measured fluorine resonance from PFCE emulsion and trifluoracetic acid (TFA) reference standard. Simultaneously acquired (d) ^1^H and (e) ^19^F images of the rabbit knee post injection of the PFCE emulsion. Reproduced with permission from Ref. [118].

**Figure 3:**
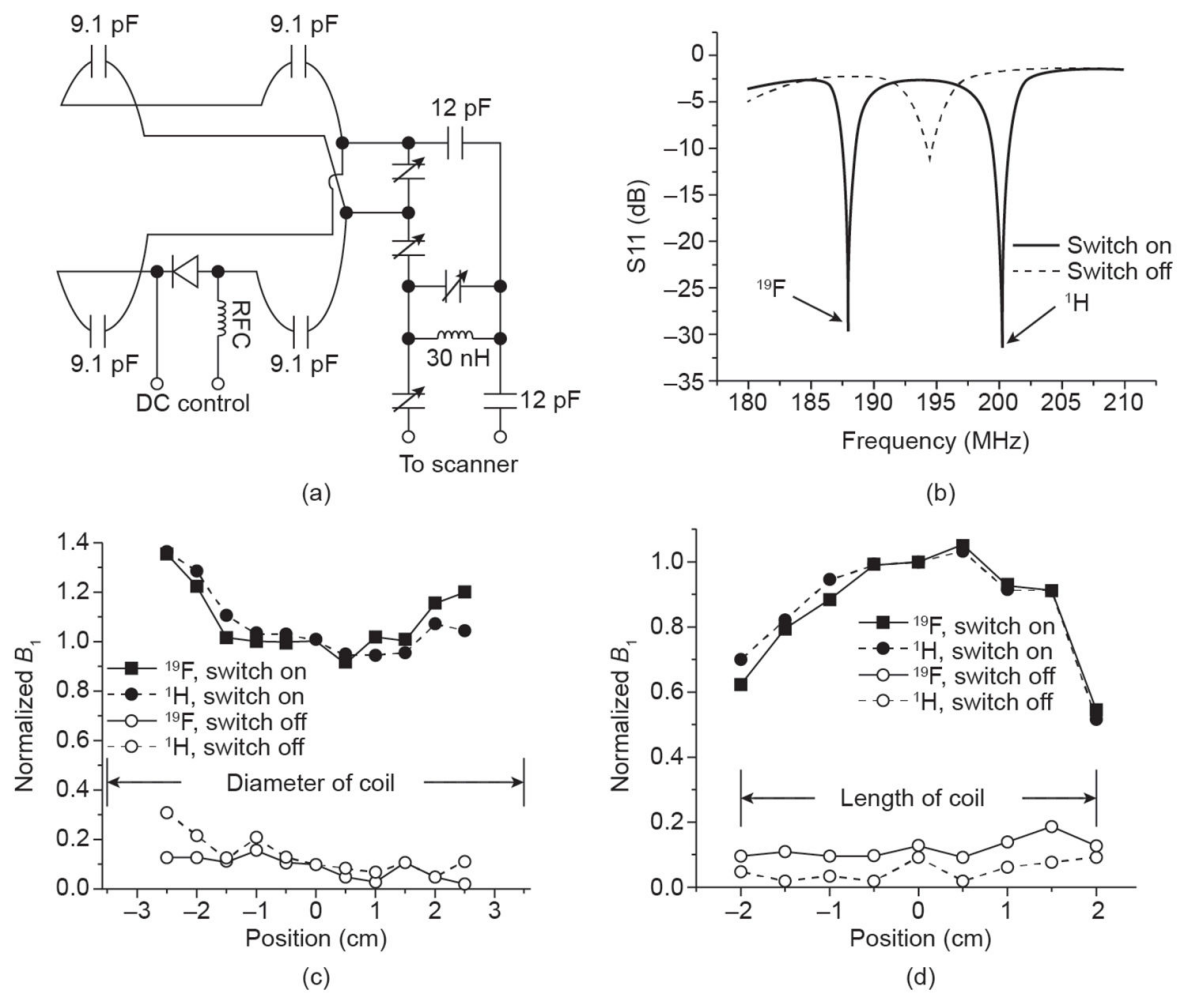
(a) Circuit design of a ^19^F/^1^H dual-frequency actively decoupled transmit-only saddle coil for a mouse image at 4.7 T. (b) S11 of actively decoupled dual-frequency saddle coil when switched on and off. (c, d) Normalized *B***_1_** field along the diameter and axis of the actively decoupled dual-frequency saddle coil when switched on and off. Reproduced with permission from Ref. [117].

A more progressive approach was proposed by Hockett et al. [118], who employed a tuned and matched dual-frequency coil that used an enclosed solenoid geometry reported by researchers at Philips Research [120], but that was modified to meet the operational constraints imposed by the size and access requirements of an arthritic rabbit-knee model (Figures 1 and 2). This design extended the dual-frequency coil simultaneous image concept to include a double-tuned circuit with an input parallel resistor, inductor, and capacitor (RLC) auxiliary resonant circuit in series with an open-coil design based on a geometry proposed by Ballon et al. [121] and Jin et al. [122]. The open-coil design allowed a cylindrical shape and multiple loops, with current steering similar to a birdcage design, in order to provide a larger coil volume with enhanced field uniformity while maintaining adequate sensitivity to support transmit-and-receive imaging of the rabbit knee at both nuclei at 3 T. A unique strength of this design was its ability to image both anatomy and exogenous contrast truly simultaneously, eliminating the chance for image registration problems while maximizing throughput. Subsequently, Hu et al. [117] extended the coupled resonator model approach of Hockett et al. [118] to a universal design technique affording the fabrication of ^19^F/^1^H dual-frequency coils in diverse configurations. For these approaches, the matching required for a dual-frequency coil to function properly with the differential impedances at the two resonant frequencies was a critical design challenge. Hockett et al. [118] and later Hu et al. [117] showed that a series capacitive matching network was theoretically effective in matching the coupled resonator to 50 Ω for both ^19^F and ^1^H frequencies. Hu et al. implemented this ^19^F/^1^H dual-frequency birdcage RF coil for *in vivo* imaging at 4.7 T to illustrate its feasibility (Figure 3). They demonstrated rapid acquisitions with a high SNR saddle coil using an actively decoupled surface coil on ^19^F frequency for both ^19^F and ^1^H signals. They achieved homogeneous excitation with high sensitivity for ^19^F MRI, while retaining sufficient SNR from ^1^H signal for anatomy images.

Recently, ^19^F/^1^H dual-tuned coils were extended by Ji et al. in Niendorf’s lab for human application at 7.0 T [116]. A modular eight-channel ^19^F/^1^H transceiver RF coil array for the human knee was designed and tested that enabled ^19^F localization of an epicutaneously applied ^19^F-NSAID ointment. In-plane spatial resolution was very high (1.5 mm × 1.5 mm) and acquisition was rapid—about three minutes of scan time. This eight-channel high-sensitivity array was adaptably positioned close to the patient and allowed for transmit field ( $B_{1}^{+}$) shaping by adjusting the magnitude and phase of different channels to minimize inhomogeneity at this ultrahigh frequency.

## 5 MR ^19^F PFC imaging

While high specificity for ^19^F detection comes from the virtual absence of background ^19^F in the body, this same paucity of signal complicates image formation optimization and motion assessments. A typical MR scanner automatically performs operator-independent preparation steps to determine the appropriate power setting for a desired flip angle, RF coil, and patient combination. Such power optimization requires rapid detection of a robust signal. The low SNR of ^19^F images precludes this essential power optimization step for single ^19^F nuclei coils.

For better simultaneous ^19^F/^1^H MRI on a clinical 3 T scanner, Keupp et al. [114] devised a novel architecture with modified spectrometers and software for concurrent dual-nuclei MRI, while other components of the scanner (e.g., the gradient system and magnet) remained unchanged. Separate transmitter and receiver devices for the non-proton nucleus were added in order to achieve simultaneous excitation of ^1^H and ^19^F nuclei. Waveforms for the prescribed RF pulses were created independently for each nucleus and fed simultaneously into the RF amplifier. With this instrumentation in particular, power optimization of the ^19^F/^1^H dual-tuned coils for a specific patient can be performed by using the rich ^1^H signal and then applying the results for both ^1^H and ^19^F imaging [123]. The spatial inhomogeneity of RF coil sensitivity (which is present in all MRI coils, and is often corrected “behind the scenes” in clinical scanners) can be applied from the ^1^H to the ^19^F channel in these dual-tuned coils [73]. For absolute quantitation of the ^19^F signal, as for serial clinical molecular imaging, these calibration and correction steps will be required.

The scan times for ^1^H scans can be quite low due to the abundance of tissue water, whereas the paucity of the ^19^F signal must be compensated with extended signal averaging. This makes the acquisition prone to motion artifacts arising from anatomical and patient motion movement, which lead to signal degradation due to blurring and ^19^F/^1^H image misregistration. Simultaneous readout of both ^1^H and ^19^F affords motion artifact correction using the ^1^H image data that is then applied to the ^19^F images [114]. With 3D radial k-space filling, under-sampling ^1^H images (i.e., reconstructed at a much higher temporal resolution than the full ^19^F k-space) allow data motion correction over prolonged image-acquisition times, which increases ^19^F SNR and quantification accuracy [114].

Fluorine detection is technically challenged by compounds with short T2 relaxation times varying across their spectral peaks and CS artifacts. To address these issues with multinuclear MR, Mastropietro et al. [124] optimized the sequence parameters of fast spin echo (FSE/RARE). Although the approach was effective for some ^19^F molecules, compounds with unique spectral properties required individual parameter tuning to the local environment. For multispectral compounds such as PFOB, common ^19^F resonant groups can be selected for acquisition at the expense of the diminished SNR efficiency incurred by ignoring other spectral lines of the compound. To capture full spectral information, a technique of chemical species separation by iterative decomposition with echo asymmetry and least-squares estimation (IDEAL) was studied, but this technique necessitates complex δ*B*_0_ correction [125, 126]. To capture signal from all PFOB spins, echo time (TE) encoding with relaxation correction has been implemented [127] in addition to pulse-phase encoding (PPE) [128]. Interestingly, chemical-shift-independent techniques like fluorine ultrafast turbo spectroscopic imaging (FuTSI) were developed and shown to capture the entire ^19^F spectrum, albeit with a significant acquisition time penalty [129].

**Figure 4:**
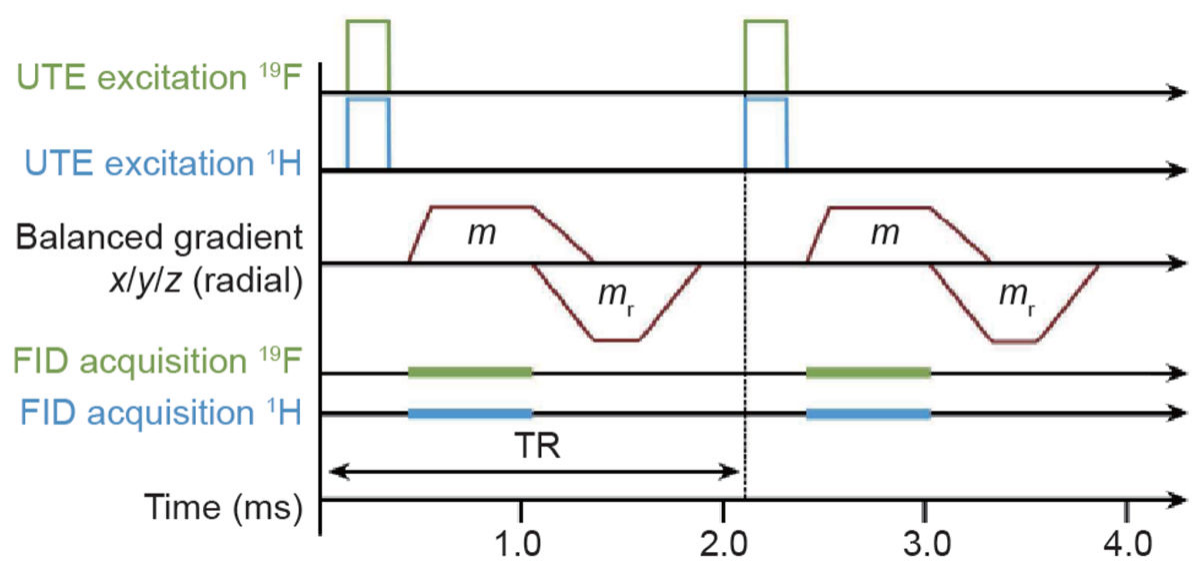
A simultaneous 3D ^19^F/^1^H UTE-SSFP pulse sequence It consists of simultaneous ^19^F/^1^H RF excitation and subsequent FID acquisition at an ultra-short echo time, using balanced gradients (*m*, *m*_r_) with a Wong-type radial readout trajectory. Reproduced with permission from Ref. [111].

A more straightforward method to image complex ^19^F spin systems, in consideration of destructive phase interference, is to acquire the signal before the spins de-phase, as in ultra-short echo time (UTE) imaging [130]. UTE potentially boosts ^19^F SNR by capturing an NMR signal before line de-phasing and significant transverse relaxation can occur [109]. Balanced steady-state free precession (SSFP) is a technique in which each gradient pulse within one repetition time (TR) is compensated by a gradient pulse with an opposite polarity, resulting in a single, re-phased magnetization vector [131]. As such, the SSFP sequence retains much of the initial magnetization (*M*_0_), which yields a steady-state MR signal with high achievable SNR. Balanced SSFP sequences have been used effectively for imaging mesenchymal cells labeled with the CellSense™ ^19^F PFC probe, which has a single resonance peak [132]. To achieve highly sensitive detection of multi-resonant imaging labels like PFOB, a dual-frequency ^19^F/^1^H UTE-balanced SSFP pulse sequence with 3D radial readout was employed on a clinical 3 T scanner to image PFC (Figure 4) [111]. The majority of the PFOB fluorine nuclei (12 of 17) are located in the CF_2_ resonances within a 1 kHz CS range that can be captured within a 90 μs TE of the UTE-SSFP sequence. This short TE preserves the NMR signal from significant de-phasing and avoids the destructive superposition of these resonances. The SNR gain achieved by constructive addition of the CF_2_ lines more than compensates for the loss in SNR efficiency imposed by 3D radial sampling (25%) and the free induction decay (FID) readout.

The UTE-SSFP technique offers several benefits for the multinuclear imaging of non-proton agents. When ^19^F contrast agents are bound to target tissues, they can exhibit further reduced T2 relaxation due to decreased molecular motion [113]. The balanced SSFP approach contributes high SNR for imaging labels with unfavorable relaxation conditions for gradient-echo methods due to *M*_0_ saturation exhibiting a long T1 and short T2. By altering offset frequency and excitation bandwidth for a particular ^19^F line group of interest, UTE-SSFP can be customized to the probe of choice. Furthermore, the UTE-SSFP sequence can be combined with simultaneous dual-nuclei techniques. Once the complex spectral signal is acquired with this sequence, the 3D radial-filled k-space data are directly reconstructed without post-processing, in contrast to CS imaging. The 3D radial dataset offers multi-resolution reconstruction, allowing analysis of the ^19^F and ^1^H data at different spatial resolutions as well as the opportunity to motion-correct the non-proton signal with temporal sub-sampling of the ^1^H data [114].

UTE-SSFP may not be ideal for all PFC probes, however. For agents with single resonance peaks, such as PFCE or PFPE, decreased SNR efficiency may result from the 3D radial k-space sampling and FID readout. Furthermore, UTE-SSFP requires that the particular line group selected must occur within a 1–2 kHz bandwidth for appropriate spatial resolution of the 3D radial readout with standard gradient systems. For PFOB, the ^19^F line group of interest spans a 1 kHz bandwidth, but other probes can differ. For compounds with fewer ^19^F nuclei than typically associated with PFC emulsions, particularly when imaged at higher than current clinical field strengths in order to achieve SNR, alternative approaches to UTE imaging such as zero time echo (ZTE) may be optimal [109]. Regardless, ultrafast imaging capability on modern MR instrumentation has enabled a renaissance in ^19^F imaging that was impossible a decade ago.

## 6 ^19^F cell tracking and macrophage labeling

The concepts of tracking cellular fates *in vivo* following the uptake of PFC emulsions labeled *ex vivo* or *in situ* have been well reviewed but deserve some recapitulation, since this concept has progressed from the bench to the clinic within the last several years [93, 99]. Phagocytic uptake of systemically injected PFC nanoparticles is an inherent feature of their natural clearance from the body by the MPS. For *in vivo in situ* labeling, relatively large volumes of particles are intravenously introduced, whereby engulfment ensues by various subgroups of leukocytes, predominantly monocytes, macrophages, neutrophils, and dendritic cells (DCs). The ^19^F-labeled leukocytes, often resident in the spleen, are recruited by cytokines into inflamed areas where their accumulation can be monitored noninvasively via MR. This concept of *in situ* PFC labeling of macrophages and the M PS organs was appreciated in the early studies detecting ^19^F hotspots in rat tumors and abscesses [133] and PFC emulsions were later detected in myocardial infarcts and cancers, although the imaging approaches were not exclusive to ^19^F MR and included US and CT [5, 35–37, 134–136]. Furthermore, studies irradiating the spleen to reduce its phagocytic function demonstrated decreased particle uptake and offered early evidence of the organ’s important MPS role in PFC particle clearance [137]. Subsequently, ^19^F MRI with *in situ* cell labeling has been used to monitor inflammation associated with autoimmune disease in experimental allergic encephalomyelitis (EAE) [67], human pancreatic cells [138], ischemic myocardium and the cerebrum [139], LPS-induced pulmonary inflammation [140], allograft rejection [96], neuroinflammatory peripheral nerve disease [141], and collagen-induced arthritis in rodents [97]. Moreover, these studies point to the qualitative relationship between the severity of the inflammation and the magnitude of the ^19^F MRI signal. Indeed, this concept is well established, and is perhaps preferable in some respects to iron-oxide-based cell-tracking techniques. Iron oxides are very sensitive markers of cells, but they tend to reduce soft-tissue resolution due to superparamagnetic bloom artifacts, which are avoided with ^19^F. The lack of magnetic artifact allows tissue and cellular boundary image detail to remain.

In many reports, specific cells such as DCs [94] and stem cells [63] have been labeled *ex vivo* and then injected or implanted *in vivo*. ^19^F imaging has been performed acutely in order to demonstrate the technique and even show the potential for using different PFCs to differentiate cell types simultaneously as well as longitudinally to follow their migration. When labeling cells *ex vivo*, the specific lineage and ^19^F cell-labeling efficiency is known and can be used to improve the biological and quantitative interpretation of the resultant data. In contradistinction, *in situ* labeling allows the ^19^F signal to be qualitatively followed but precludes accurately knowing which cell types are being imaged or quantitatively related to the ^19^F signal intensity on spin-density weighted MR techniques.

Recently, Ahrens et al. [98] used PFPE nanoparticles (CS-1000) for ^19^F cell-tracking of immunotherapeutic mature DCs in patients with colorectal adenocarcinoma. The PFPE agent used was rigorously tested for acute toxicity, cytotoxicity, and genotoxicity prior to clinical use without adverse effects at 100-fold the clinical dose anticipated. PFPE was incorporated into nonphagocytic cells without adjunctive cationic lipids and without altering the cellular phenotype. It is notable that only viable cells were labeled with PFPE. However, *in vivo* cell death can liberate the PFC, which may then be engulfed by macrophages, leading to a false positive signal. The same is true of iron oxides, and can be an inherent confounding problem of the technique in general. Post-implantation DCs were noted as “hotspots” at the injection site, and that signal diminished by 50% the next day. This result affirmed that the label cells were injected and, in two patients, the technique allowed the number of cells to be estimated. The further disposition of the cells was not appreciated. A correlation with the lack of response—whether due to an inadequate therapeutic load injected, marked early cell death, DC migration, or an ineffectiveness of the treatment concept—remains an open question. Although this clinical study used rudimentary data-acquisition methods and a suboptimal MRI detector (surface coil) design, it still showed the potential for ^19^F imaging efficacy with a clinical scanner. The advancements in coil, pulse sequence design, and motion correction are now better understood and when implemented will likely improve the diagnostic results achieved.

## 7 ^19^F PFC nanoparticle molecular imaging of thrombus

The use of ^19^F imaging and PFC nanoparticles for thrombus diagnosis dates back more than a decade. Thrombus is rich in molecular epitopes for targeting, in particular fibrin, thrombin, and in some instances, platelets. Yu et al. [142] demonstrated high-field molecular imaging of fibrin-targeted paramagnetic PFOB nanoparticles using human clot phantoms, and showed not only the ^1^H T1-weighted contrast of PFC particles decorated with surface Gd^3+^ DTPA-BOA, but also the corresponding ^19^F signal. Although the improved ^19^F R1/R2 relaxation enhancement of Gd^3+^ DTPA-BOA in the lipid surfactant was not appreciated at the time, in hindsight it is clear that this factor contributed to the robust ^19^F result.

**Figure 5:**
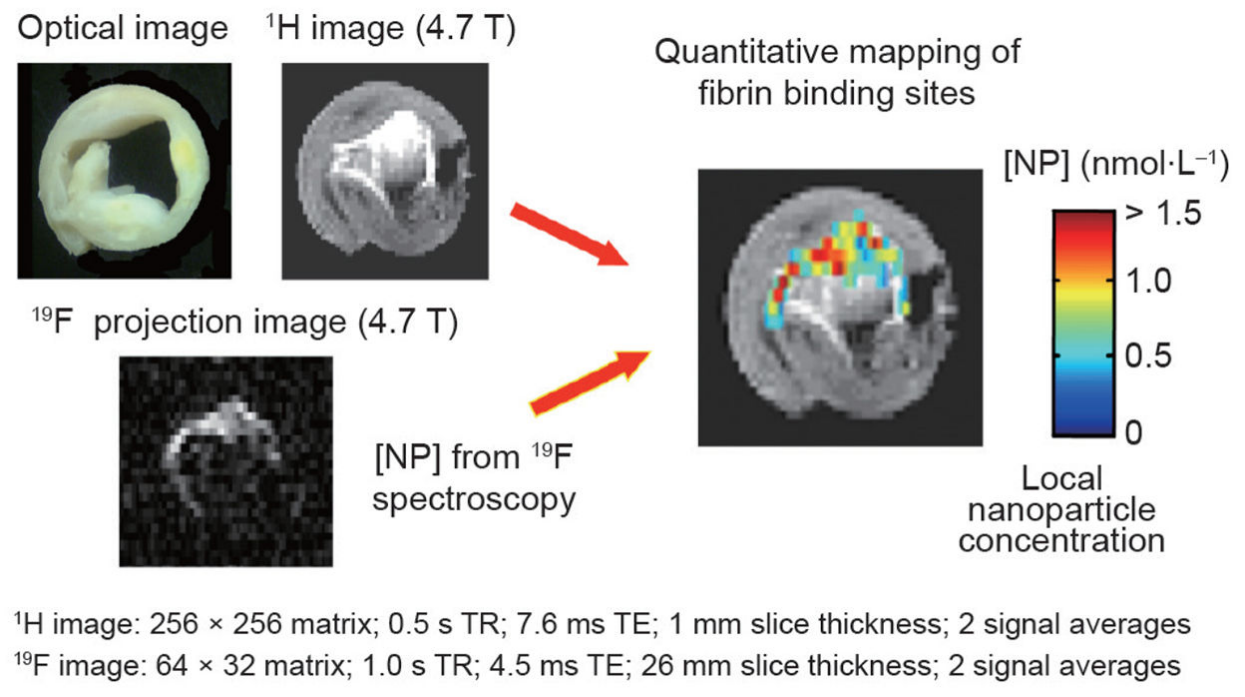
Proton and fluorine images of a carotid endarterectomy specimen obtained at 4.7 T (independently) and fused Note that the fluorine pixels superimposed on the T1-weighted contrast image confirm and quantify the fibrin-specific signal. Also note that some regions of apparent T1 brightness are not aligned with the fluorine signal, and are most likely not derived from the paramagnetic nanoparticle. (Bright spots do not necessarily indicate a contrast agent.) Reproduced with permission from Ref. [55].

In subsequent studies, the dual imaging proton and the quantitative potential of ^19^F imaging and spectroscopy with paramagnetic PFOB nanoparticles was demonstrated at 4.7 T, and was corroborated by proton relaxation rates at 1.5 T and radioactive gadolinium metal using neutron activation analysis [55]. The clinical relevance of this concept was illustrated with *ex vivo* human carotid endarterectomy for uniquely and quantitatively detecting ruptured plaque (Figure 5). The use of ^19^F spectroscopy and imaging for the simultaneous differentiation of two nanoparticle emulsions with PFCE or PFOB cores targeted to fibrin clot phantoms illustrated the potential for noninvasive phenotypic characterization of pathological targets. The underlying proposition was that pathologic differentiation of complex tissues will require multiple biomarkers, and the unique ratio of the receptor expression levels could be used to characterize and diagnose pathology. In this experiment, the PFCE nanoparticles incorporated gadolinium. ^19^F images were acquired using steady-state gradient-echo techniques and spectra acquired with volume-selective and nonselective sampling. The imaging used conventional T1-weighted imaging clots with PFCE nanoparticles, which were enhanced as expected with the intensity contrast signal decreasing monotonically with particle concentration. All clots were visualized using wide-bandwidth ^19^F imaging while a restricted bandwidth excitation permitted the independent imaging of PFCE or PFOB nanoparticles.

In the coagulation cascade, thrombin represents a principal target of direct and specific anticoagulants, which can be accessed via a thrombin inhibitor (Phe[D]-Pro -Arg-chloromethylketone, or PPACK) complexed onto colloidal nanoparticles [143]. This theranostic construct was devised as a first-in-class anticoagulant with a prolonged and highly localized therapeutic impact conferred by its multivalent thrombin-absorbing particle surface. In an *in vivo* acute arterial thrombosis model, PPACK nanoparticles outperformed both heparin and uncomplexed PPACK in inhibiting thrombosis. ^19^F MRS (11.7 T) confirmed that the PPACK nanoparticles specifically bound to sites of acute thrombotic injury. The ^19^F MRI showed co-localization of particles within an artery. It is of interest that although these thrombin-inhibiting nanoparticles only increased the systemic bleeding time for 10 min, they provided prolonged clot inhibition.

More recently, noninvasive identification of developing thrombi using ^19^F MRI and 2-antiplasmin peptide (α2^AP^)-targeted PFC nanoemulsions was reported [144]. Ligand functionality was achieved under mild coupling conditions using a sterol-based post-insertion technique. Targeted imaging of murine inferior vena cava thrombus using simultaneous acquisition of ^1^H and ^19^F MR images at 9.4 T was achieved with excellent SNR and CNR. The α2^AP^-PFCs construct was also evaluated for the diagnosis of experimentally induced pulmonary thromboembolism. α2^AP^-PFCs targeted the thrombus as clot formation progressed, but 60 min after thrombus induction, no detectable ^19^F signal was appreciated. This result suggests that crosslinking of fibrin by the thrombin-activated factor XIIIa was completed, and points to the utility of α2^AP^-PFCs for differentiating thrombolytic labile versus drug-resistant thrombus. This therapeutic approach could improve the risk-benefit balance for thrombolytic treatment.

The fluorine-rich core of PFC nanoparticles can also serve as a quantitative reference from which to normalize and characterize chemical exchange saturation transfer (CEST) agents targeted to fibrin clots or other targets [145]. CEST chelates have exchangeable protons (—NH, —OH, etc.) that resonate at a CS that is distinguishable from the bulk water signal. RF pre-pulses applied at the appropriate frequency and power can saturate the exchangeable protons that then transfer into the bulk water pool and reduce the equilibrium magnetization. PFC nanoparticles functionalized with paramagnetic CEST (PARACEST) chelates offer a switchable contrast, which is achieved by adjusting the pulse sequence parameters. This important feature of CEST agents obviates the need for pre- and post-injection images, which is common with most ^1^H-based molecular imaging contrast probes. Moreover, the newly designed PARACEST PFC nanoparticles produced a CNR of 10 at the clot surface, which helped to address the lack of contrast sensitivity of early PARACEST agents. In addition to lowering the effective detection limit by delivering very large payloads of PARACEST chelates on the PFC nanoparticle surface, the PFC core itself provided a ^19^F signal for quantitative particle-binding estimation. The simultaneous availability of two unique signatures, ^19^F and PARACEST, directly indicated the voxel concentration of the PARACEST chelates that were bound. Moreover, when bound to the clot surface, the PARACEST nanoparticles produce more than two-fold higher PARACEST contrast than when free in suspension. This was suggested to be due to reduced nanoparticle mobility and slowed water-exchange kinetics. The increased bound water lifetime lowered the detection limit from 4.13 nmol·L^−1^ for nanoparticles in suspension to 2.30 nmol· L^−1^ for nanoparticles bound to a target. Indeed, CEST and PARACEST agents have evolved from their humble beginnings to become important MR contrast agents [144, 146–178].

## 8 ^19^F angiogenesis imaging

Angiogenesis is a critical early feature of normal tissues, such as the endometrium, bone growth plates, and wound healing, as well as of pathologies such as rheumatoid arthritis, atherosclerosis, asthma, and cancers. The detection of angiogenesis via molecular imaging of the α_ν_β_3_-integrin expressed by neoendothelial cells requires low nanomolar sensitivity per voxel for detectability. High-resolution ^19^F imaging of angiogenesis was first used to detect and quantify the neovasculature in a rabbit model of aortic valve disease with α_ν_β_3_-PFC-nanoparticles [70]. Atherosclerotic New Zealand White rabbits were treated with α_ν_β_3_-targeted PFC nanoparticles or untargeted PFC nanoparticles. The excised aortic valve leaflets were thickened and inflamed. ^19^F MRS at 11.7 T of the valve leaflets from rabbits treated with α_ν_β_3_-targeted PFC nanoparticles had 220% more ^19^F signal than their counter-parts receiving untargeted PFC nanoparticles. Furthermore, in a competitive inhibition treatment group, the valve leaflet ^19^F signal was reduced by 42%, supporting the specificity of the targeting. In a second cohort of atherosclerotic rabbits, ^19^F spectroscopy performed at 3 T using a clinical scanner again demonstrated the effectiveness of α_ν_β_3_-targeted PFC nanoparticles for neovascular imaging, which was further corroborated with immunohistochemistry.

Molecular imaging of U87 glioblastoma at 7 T in mice using α_ν_β_3_-PFOB nanoparticles similarly showed concentrations in mice tumors to be greater than in the control, although nonspecific background signal originating in blood at this high-field strength persisted in this experiment [179]. These imaging results were corroborated by histology and by fluorescence microscopy. Importantly, this was the first ^19^F imaging example in brain tumor angiogenesis that was obtained with integrin-targeted PFC nanoparticles. This group later developed polymeric encapsulation of PFOB with an extended circulatory half-life, and then functionalized it with an Arg-Gly-Asp (RGD, an anti-integrin) ligand in order to demonstrate its use for tumor imaging with ^19^F as well [180, 181].

As mentioned earlier, angiogenesis is an important constituent of inflammatory pulmonary diseases, such as asthma. Early neovascular expansion in the lungs in patients is very difficult to assess noninvasively, and particularly quantitatively. With the notable exception of significant lung tumors, pulmonary ^1^H MRI of parenchymal disease is challenging due to a paucity of tissue water protons, respiratory motion, and CS artifacts. ^19^F/^1^H MR molecular imaging with α_ν_β_3_-targeted PFOB nanoparticles can be used to directly measure neovascularity. To validate the ^19^F/^1^H MRI technique in the lung, an established rat left pulmonary artery ligation (LPAL) model was employed [182]. LPAL induces the bronchial artery vasculature to proliferate into zones of acute inflammatory lung injury, notably surrounding the bronchioles and large vasculature proximal to the ligature [182].

**Figure 6:**
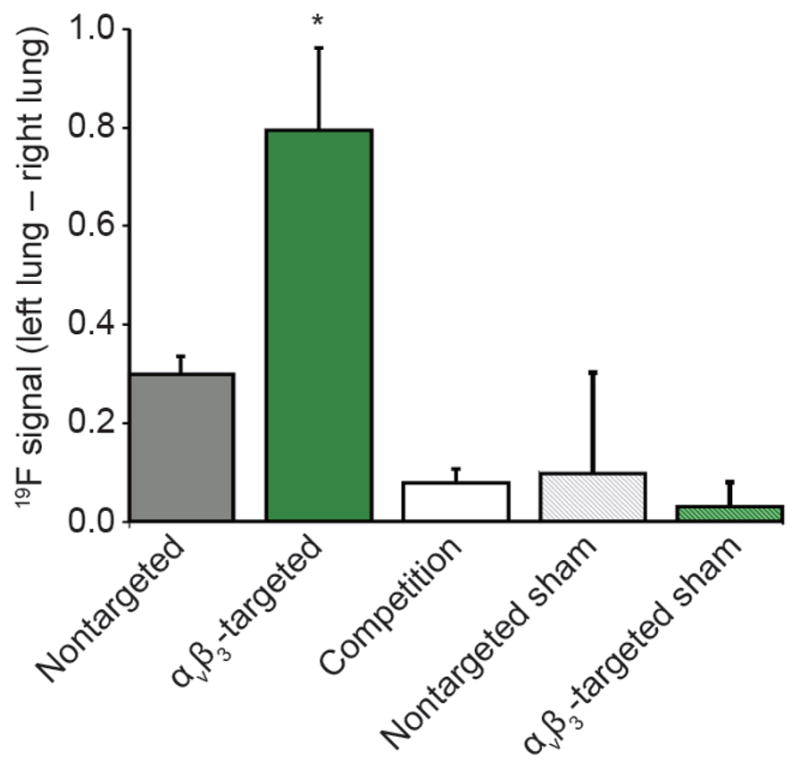
Comparison of left-right lung signal in day 3 LPAL rats and sham-operated rats, administered nontargeted, α_ν_β_3_-targeted, or competitive-inhibited α_ν_β_3_-targeted PFC nanoparticles after two hours LPAL rats receiving α_ν_β_3_-targeted nanoparticles had the greatest ^19^F left lung signal compared to the nontargeted PFC particles (*P* = 0.005). Competitive inhibition with α_ν_β_3_-targeted oil nanoparticles decreased the lung ^19^F signal (*P* = 0.0001). The ^19^F signal in sham-operated control rats was also greatly decreased (*P* = 0.002). Reproduced with permission From Ref. [183].

**Figure 7:**
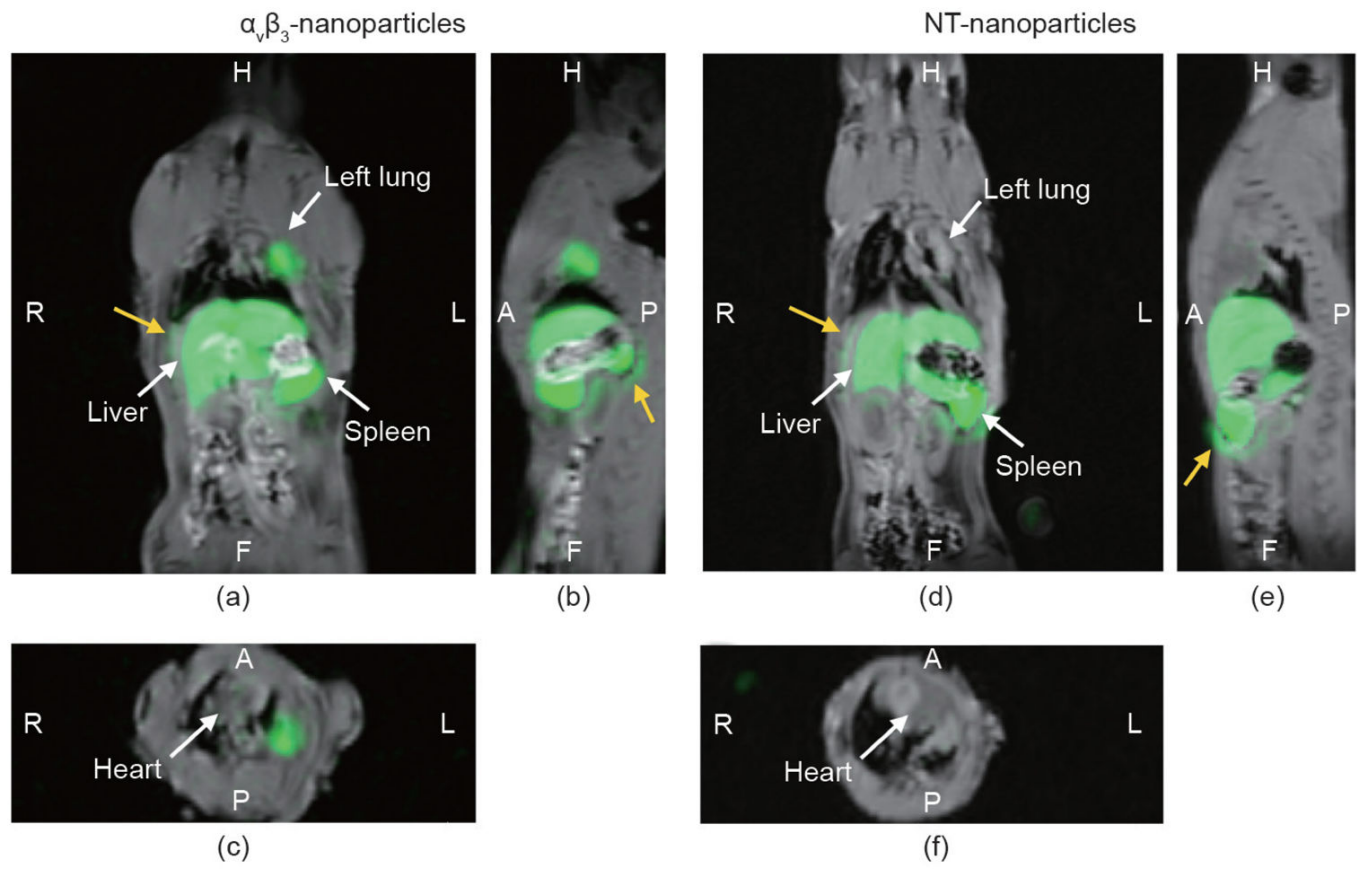
Whole-body ^19^F/^1^H images of a rat thorax and abdomen two hours after the injection of α_ν_β_3_-targeted or nontargeted (NT) PFC nanoparticles (green) (a) Coronal plane of the rat following α_ν_β_3_-targeted nanoparticles, showing accumulation in the left lung adjacent to the heart, and reticuloendothelial (RES) clearance of particles in the liver and spleen. Little signal was detected in the right lung. (b) Sagittal view of the targeted nanoparticle accumulation in the left lung. (c) Transverse view at the level of the heart, showing bound nanoparticles in the left but not in the right lung. (d) Coronal plane of the rat following NT-nanoparticles, showing no visual accumulation in the injured left lung adjacent to the heart, but with significant RES clearance of particles into the liver and spleen. (e, f) Sagittal and transverse views of the rat thorax following NT-nanoparticles showing negligible accumulation in the injured lung. No signal is appreciated in the right lung. In all these images, no off-target contrast was appreciated in the surrounding musculature. The ^1^H image shown above was obtained at higher resolution using a clinical 3 T scanner for higher thoracic anatomical clarity. Yellow arrows indicate examples of ringing artifact recognized in this particular MRI sequence, which are common to high-signal areas (i.e., liver and spleen). Reproduced with permission from Ref. [183].

Three days after pulmonary artery ligation, simultaneous ^19^F/^1^H MRI at 3 T was performed following the injection of α_ν_β_3_-targeted PFC nanoparticles [183]. The injured rat lung and bronchi had an increased ^19^F signal with targeted PFC particles compared to the nontargeted agent (Figure 6). Almost no ^19^F signal was appreciated in the control right lungs with targeted or nontargeted PFC nanoparticles. Competitive inhibition of α_ν_β_3_-targeted PFC nanoparticles decreased the ^19^F angiogenesis signal in the ischemic left lung, a reduction that was not different from the ^19^F signal obtained in the nontargeted group (Figure 7). Supporting fluorescent and light microscopy illustrated the heavy nanoparticle decorating of vessel walls in and around the large bronchi and large pulmonary vessels. These results demonstrated that ^19^F/^1^H MR molecular imaging with α_ν_β_3_-PFC nanoparticles provided a direct and reproducible readout of neovascularization in the injured lung.

**Figure 8:**
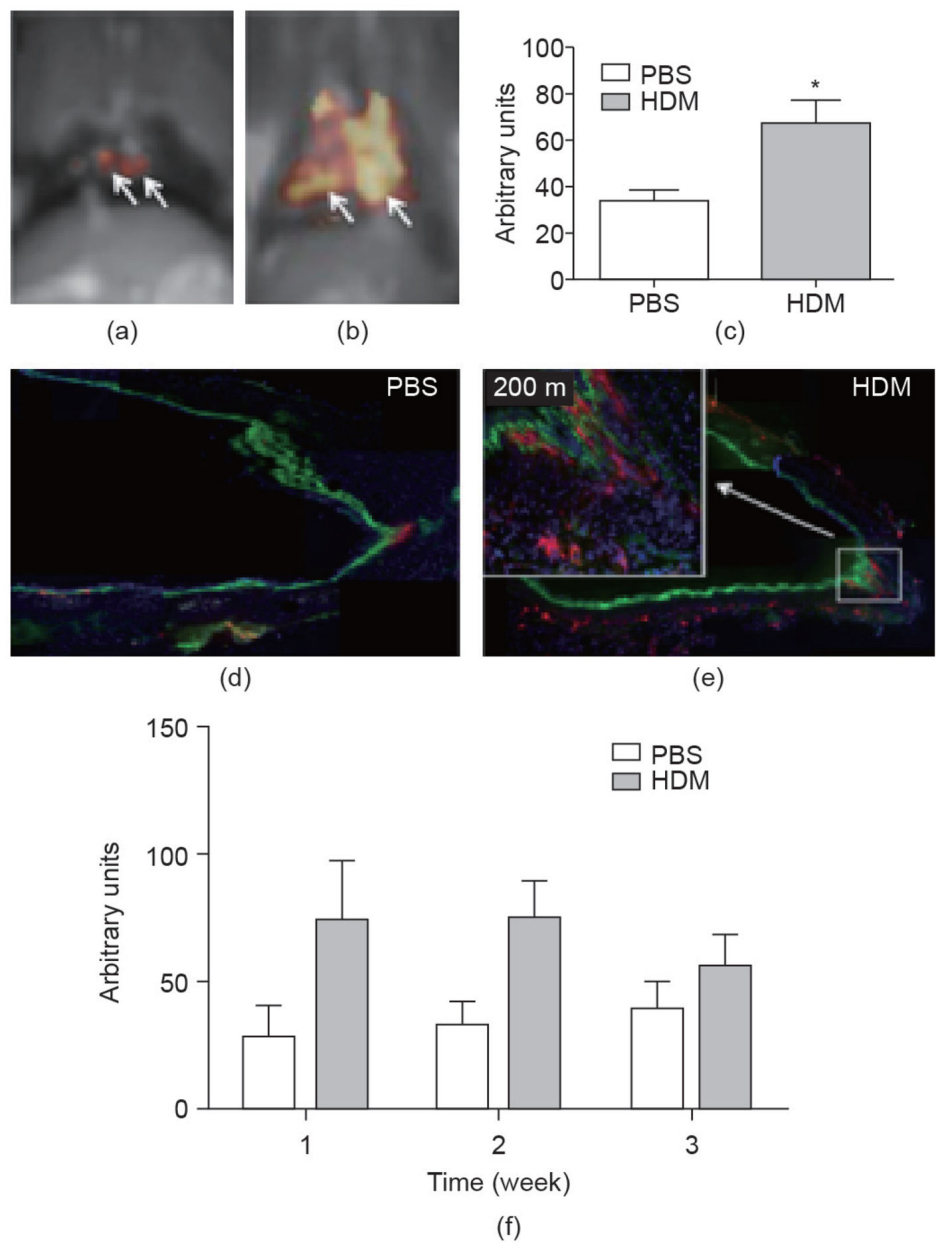
(a, b) ^1^H/^19^F dual simultaneous MR molecular imaging scans of PBS and HDM rats two weeks after the onset of sensitization using α_ν_β_3_-targeted PFC nanoparticles. Note that uniform ^19^F weighting was applied to all images from all animals, and voxel size was set to the original acquisition of 1.25 mm × 1.25 mm × 1.25 mm. (c) The overall three-week effect of HDM sensitization on total ^19^F signal in arbitrary units compared to PBS controls (*P = 0.007). (d, e) Rhodamine labeled α_ν_β_3_-targeted PFC nanoparticles in frozen sections of large bronchi of PBS and HDM rats two weeks after the onset of sensitization. (f) Sub-analysis weekly comparisons of the effect of HDM sensitization versus the PBS controls, showing early induction of angiogenesis by week one with a statistical peak difference observed after two weeks between PBS and HDM treatment (*P* = 0.05), which was followed by a neovascular decline in the magnitude and treatment difference at three weeks. Reproduced with permission from Ref. [184].

Next, this validated ^19^F imaging technique was applied to interrogate the aberrant bronchial neovascularity associated with chronic asthmatic airway as a possible factor contributing to persistent airway wall edema and sustained leukocyte recruitment [184]. The causal relationships between exposure to house dust mites (HDMs) and the development of asthma is well established. Rats were exposed bi-weekly to HDM inhalation and studied after one, two, or three weeks of antigen exposure. The time course of the appearance of increased blood vessels within the airway wall was assessed noninvasively with ^19^F MRI and corroborated with quantitative microscopy. A methacholine challenge study was used to assess for hyper-reactive airway response, characteristic of asthma. After three weeks of HDM exposure (six inhalation doses), the number of mature vessels counted within the airway walls of bronchial airways (0.5–3 mm perimeter) increased significantly. These vascular changes were accompanied by increased airway responsiveness to methacholine. However, after only one or two weeks of the HDM challenge, no significant change in functional vessel numbers or methacholine airway reactivity was noted. ^19^F/^1^H MRI at 3 T with α_ν_β_3_-targeted PFC nanoparticle infusion established increases in ^19^F signal in rat airways after one and two weeks of bi-weekly HDM, revealing the earlier activation of the process of neovascularization before increased airway hyper-reactivity was appreciated (Figure 8). Moreover, when gross pathological and clinical manifestations were manifest, ^19^F molecular imaging showed that the acute neovascular expansion phase had passed.

This use of ^19^F/^1^H for pulmonary imaging is particularly relevant because MRI is not clinically exploited for interrogating diffuse parenchymal lung diseases, yet the prevalence of these pathologies and healthcare costs to treat them are soaring. Although CT is often used to study lung pathology, in general it has a low sensitivity to early disease, where medical management can have the greatest impact. Moreover, for patients dealing with lifetime chronic diseases, serial exposure to ionizing radiation is problematic, particularly for patients who are diagnosed at younger ages. Echocardiography to assess right ventricular (RV) function, morphology, and valvular hemodynamics is often used as a surrogate marker for increasing pulmonary hypertension, but it is imprecise, relatively insensitive, and provides no direct quantification of the spatial extent or distribution of the disease in the lung itself. Indeed, simultaneous ^19^F/^1^H MR molecular imaging at 3 T with the improvements in coils and imaging techniques now offers high potential to address these inflammation-based typically fibrotic diseases when the cadre of new medicants can be useful and optimized to the individual.

## 9 Conclusions

This introduction to ^19^F MRI has illustrated how a concept first considered in the 1970s is now on the verge of clinical relevance in 2015. Improvements in MR instrumentation, coils, and pulse sequences have begun to overcome the fundamental issues that have precluded clinical translation for many years. Whether for cell or targeted molecular imaging or for other applications such as oxygen sensing [185–190], PFC particles are coming to the fore in MR by virtue of their dense fluorine content and relatively bio-inert properties, offering imaging possibilities that take advantage of and complement traditional ^1^H MRI. While more work needs to be done, it seems that a breakthrough application is near, and once that is achieved, the floodgates of applications for these unique nuclei will be open. The only barrier that seems to stand between this technology and its clinical reality is unmitigated will power.
